# Review: understanding the properties of amorphous materials with high-performance computing methods

**DOI:** 10.1098/rsta.2022.0251

**Published:** 2023-07-10

**Authors:** J. K. Christie

**Affiliations:** Department of Materials, Loughborough University, Loughborough LE11 3TU, UK

**Keywords:** amorphous materials, high-performance computing, glass, molecular dynamics

## Abstract

Amorphous materials have no long-range order in their atomic structure. This makes much of the formalism for the study of crystalline materials irrelevant, and so elucidating their structure and properties is challenging. The use of computational methods is a powerful complement to experimental studies, and in this paper we review the use of high-performance computing methods in the simulation of amorphous materials. Five case studies are presented to showcase the wide range of materials and computational methods available to practitioners in this field.

This article is part of a discussion meeting issue ‘Supercomputing simulations of advanced materials’.

## 1. Introduction to amorphous materials

A full description of the structure and properties of amorphous materials is beyond the scope of this paper, but see e.g. [1,2] for more discussion. We will use the definition that an amorphous material is one which has no long-range structural order. This lack of order has important consequences: without long-range order, the material has no unit cell, no reciprocal lattice, no Bragg peaks in its diffraction pattern. A substantial part of the formalism used to study more ordered, crystalline materials is no use.

The biggest class of amorphous materials are glasses, and the words ‘glass’ and ‘amorphous material’ are often used synonymously, although this is not quite correct, as there are materials which are structurally amorphous (such as amorphous silicon (a-Si)) which do not undergo a glass transition.

Amorphous materials are disordered, but their structure is not random, and is still governed by the physics and chemistry present in the crystalline counterparts. It is common, for example, for the nearest-neighbour bond lengths to be similar in amorphous and crystalline phases of the same material, but for more disorder to arise in the distribution of bond angles, and at larger length scales.

Glasses are often described by the ‘continuous random network’ model [3], first formulated by Zachariasen. His rules for glass formation for oxide glasses state that the cations must have low coordination numbers (typically 3 or 4), sitting at the centre of polyhedra with the negatively charged oxygen anions at the corners. No oxygen atom can be bound to more than two cations, and those which are bound to two are known as bridging oxygen (BO) atoms, while those that are bound to less than two are known as non-bridging oxygen (NBO) atoms. The number n in the $Q^{n}$ distribution refers to the number of BO atoms bound to a glass-forming cation (also known as a network former, examples including Si, P and B), and the network connectivity (NC) of a glass is the average value of n.

Oxides which cannot form glasses on their own, for example ${\mathrm{Na}}_{2}O$ and CaO, can be a component of glassy systems, but these cations violate the rule of low coordination number so do not contribute to the network directly, and are almost completely surrounded by NBO atoms; they are known as network modifiers, and can be distributed homogeneously in the glass, but can also cluster, leading to a material with different properties in different regions [4].

We can draw a distinction between short-range order (SRO) and medium-range order (MRO) [1]. There is no sharp dividing line, but broadly SRO is the order present in the first one or two nearest-neighbour distances, and MRO is the order at distances of approximately 2–20 nearest-neighbour distances. It is very common for an amorphous material to have strong SRO in the form of narrow distributions of nearest-neighbour bond lengths and angles, but the amount of MRO varies by material much more.

A very common way to prepare glasses experimentally is through the ‘melt-quench’ method [1]. The components of the glass are mixed together, and heated to a temperature above the melting point of the composition. The mixture is then cooled quickly and the material settles into a disordered (amorphous) solid state. To make a glass in this way, the cooling must occur quickly enough that the material cannot find its lower-energy crystalline state. The ease with which it can do this varies between materials.

Other methods of making a glass include the sol-gel method [5], in which the glasses are built up from smaller molecules, which condense together to form a porous structure, often containing substantial amounts of water. Crystalline materials can also be made amorphous through the application of pressure [6], or through bombardment by radiation.

## 2. Molecular dynamics simulations of amorphous materials

To simulate amorphous materials, the most common method is to mimic the experimental ‘melt-quench’ preparation, with a molecular dynamics (MD) simulation [7]. In MD simulations, the positions of the atoms are individually defined, and at each time step, a computation of the interatomic forces is made. The atomic positions are updated by F=ma, where F is the atomic force on an atom of mass m, which subjects it to an acceleration a, before computing the interatomic forces in their new positions, which starts the next time step. By starting this simulation at a temperature above the melting point $T_{m}$, and cooling it down through the glass transition temperature $T_{g}$ to the temperature of interest (typically room temperature), an approximation to the experimental ‘melt-quench’ approach can be achieved.

The success or otherwise of this approach is strongly dependent on how well the interatomic forces are modelled, and there are several ways of doing this. The most accurate methods with which one could plausibly run an MD simulation are those methods based on density-functional theory (DFT) [8], in which the quantum-mechanical nature of the interatomic forces is explicitly included. These simulations, although accurate, are very computationally expensive, and limited, therefore, to small length- and timescales.

It is possible to approximate the interatomic forces through the generation of empirical expressions, usually called potentials. These expressions are deliberately designed to be computationally cheap to compute, at the expense of some accuracy, but they are, therefore, accessible to larger length- and timescales than DFT simulations. As they do not involve quantum-mechanical behaviour, they are often referred to as ‘classical’ potentials.

There is a hierarchy of potentials; as they become less complicated, they become less accurate, but also less computationally expensive. The investigator must decide for themselves where in the trade-off between accuracy and expense they need to be to extract the information they need from the simulations. Probably the most complex classical potentials are the machine-learned (ML) potentials [9], which do have an analytic form, albeit a very complicated one. Less complex are potentials for metals, such as those based on the embedded-atom techniques [10,11], which are explicitly many-body in forms. Non-metallic amorphous materials are often represented with polarizable potentials [12,13], in which the oxide anions (and possibly others) are represented as polarizable. It has been shown that the use of the shell model to incorporate polarizability is vital to get the correct NC and $Q^{n}$ distribution which control the *in vivo* activity of bioactive glasses, for example [14]. The simplest and cheapest potentials are rigid-ion, in which the atoms are represented as simple points.

The scope of this review is limited to those simulations which require high-performance computing (HPC) facilities to carry out, and I define this scope here. All but the very smallest DFT-based MD simulations will require HPC facilities. Data from DFT, typically in the form of energies, interatomic forces and stresses, are often used as a training set to fit classical potentials of all types, in the hope of getting a potential which is computationally cheap to compute but very accurate. In that sense, potential fitting is also dependent on HPC. As we go down the hierarchy of potentials, it is easier to conduct the simulations on smaller computing facilities, but HPC simulations are still required for large-scale simulations, as we will see.

## 3. Methodologies and computing observable quantities

As above, an amorphous material does not have any long-range structural order; therefore, it has no unit cell. It is usual, therefore, to begin the simulation with a cell containing the appropriate amount of atoms at some experimental density, above the melting temperature. The atoms are typically randomly arranged, usually with a constraint that they not be too close (as in e.g. [15]), to prevent unphysical configurations which might crash the simulation at the start. Melting from the crystal structure directly can also be done, but this can lead to unphysical remnants of the crystal structure still present in the glass. The cell is usually subject to periodic boundary conditions. The lack of long-range order usually means an amorphous material is isotropic, so for computational convenience, the simulation cell is often cubic.

The simulation cell is equilibrated at this high temperature, using either NPT or NVT ensembles, and then cooled through a series of temperatures until the solid state is formed.

The cooling rate is a well-known problem in the simulation of glasses, either DFT or classically. The cooling rate during an experimental quench of a glass depends on the material used, but would typically be approximately $1\text{–}1000\;{K\;s}^{-1}$ for oxide glasses, and perhaps as high as $10^{6}\;{K\;s}^{-1}$ for metallic glasses. This is substantially slower than typical cooling rates obtained during simulations, for which $10^{11}\;{K\;s}^{-1}$ would be a good cooling rate for a classical simulation [16], and for which DFT simulations hover around $10^{13}\;{K\;s}^{-1}$ [17]. There is evidence that at least the short-range structure of an amorphous material is well converged at cooling rates as high as $10^{12}\;{K\;s}^{-1}$ [18,19], but this is typically slower than cooling rates achieved in DFT simulation, and the medium-range structure of glass may need slower cooling rates still [20].

Mixed approaches, e.g. using classical MD to perform the quenching part of the simulation before swapping to DFT for the conditions of interest, have been used with some success. Caution must be employed, however, as the system will solidify as it goes through $T_{g}$ with the structure dependent on the potential being used at that time. This structure will be ‘baked in’ to the simulation as the simulation is too short to allow for large-scale rearrangements at low temperature, and if this structure is less accurate, this lack of accuracy will also occur in the room temperature simulations.

Thapa & Drabold [21] identify four potential problems with DFT simulations of glasses, including the cooling rate. The other three are their small size, the effects of PBCs, and whether it is appropriate to use only the Γ point of the Brillouin zone for computations. There is little that can be done about any of these last three, short of using more computational resources.

The only way to verify a computer simulation of any material is by comparison to data obtained experimentally. In amorphous materials, the two most common experimental techniques by which this is done are diffraction (either X-ray or neutron), and solid-state nuclear magnetic resonance (NMR) measurements.

The positions of all the atoms are known at all times in simulation, so the pair-distribution function g(r) or the diffraction pattern S(k) (which are related to each other via a Fourier transform) can be computed and directly compared; a quantitative value on how close the agreement is can be obtained through computing the $R_{x}$ parameter [22]. From simulation, it is easy to decompose these into partial functions, related to two species in a multicomponent glass, and this can be compared with neutron diffraction spectra obtained through e.g. isotopic substitution experiments [23]. Such approaches have led to elucidating the distances over which chemical ordering can be found in glass, in some cases many tens of Angstroms [24]. Comparison with solid-state NMR spectra is also very useful, and this is discussed in one of the examples below.

It is also straightforward to compute, from simulation trajectories, structural properties which give insight into the simulation, but which are challenging or impossible to access from experiment. These include bond-angle distributions (e.g. [25]), and topological properties such as the distribution of ring sizes.

Owing to their structural disorder, amorphous materials often have lower conductivities than their crystalline counterparts. Amorphous metals are often electrically conductive, but other amorphous materials, e.g. oxides, are usually insulators or semiconductors. The electronic density of states can be computed down to the level of orbitals [26], as for crystalline materials, and the computation of the electronic structure has the same advantages and disadvantages, e.g. the known effect of different functionals on the value of the band gap.

The use of PBCs allows for the study of bulk glasses, but one can also turn off PBCs in one direction to allow for the study of the surface of glasses, or the glass interaction with, for example, water or biomolecules [27]. Often the surrounding environment changes the surface chemistry of the glass, for example, by hydroxylating dangling bonds on a silica surface [28].

## 4. Applications

In this section, I describe some specific studies where the use of HPC has illuminated some aspect of the study of amorphous materials. I make no pretence that this section includes every piece of work in the field, and I have chosen these examples based on two criteria: (i) they are active fields of research at the time of writing and (ii) taken as a whole, the examples illuminate the broad range of amorphous materials and HPC methods being studied and used.

### (a) Charge trapping in amorphous oxides

The use of thin films of amorphous oxides is common across a very wide range of devices, including solar cells, superconductors, transistors and photocatalysts, among others [29]. These films can be polycrystalline, in which case understanding the structure of the grain boundaries is crucial to understand the material’s behaviour [29], or they can be amorphous [30]. In both cases, the electronic properties are quite different to that of the bulk material, and can be very difficult to disentangle by experiment; computer simulation has played avital role.

The study of charge trapping, where an electron or hole is localized to a region of a material, typically surrounding a defect in the material structure, presents interesting methodological challenges for the simulation of amorphous materials. Mora-Fonz *et al.* [31] state that ‘accurate prediction of polaron states is challenging due to the self-interaction error inherent in DFT’. It is usual to use hybrid functionals, in which an amount of Hartree–Fock exchange is combined with another exchange–correlation functional. The use of hybrid functionals is computationally demanding, and so it would be prohibitively expensive to use them throughout the full MD trajectory, especially if multiple simulations are required to obtain accurate statistics. The approach adopted is to run an ensemble of simulations using classical MD and a full melt-quench trajectory, followed by relaxation with a hybrid functional (sometimes preceded by a relaxation with a non-hybrid functional) to give accurate electronic properties. This has been successfully applied to amorphous ${\mathrm{SiO}}_{2}$ [32], ${\mathrm{TiO}}_{2}$, ZnO [33], ${\mathrm{Sm}}_{2}O_{3}$ [34], ${\mathrm{Al}}_{2}O_{3}$ [35] and ${\mathrm{HfO}}_{2}$ [36,37], to compute e.g. defect formation energies, and activation energies for diffusion. This is a case where mixed simulation methods are being used successfully, presumably because there is little dependence of the local energies on the medium-rangestructure.

Strand *et al.* [30] emphasize that comparison with experimental results is very important, not just because of the difficulty of correctly simulating electronic structures as discussed above, but also because the disordered amorphous structure allows for a variety of atomic environments. One constant seems to be that electrons and holes are more deeply trapped in amorphous films than in crystalline ones [30].

### (b) Machine-learned interatomic potentials

In recent years, the development of ML potentials has come to the fore. This refers to the use of ML techniques to fit, for example, energies, interatomic forces and stresses calculated from DFT simulations to a ML interatomic potential. The exact functional form of this potential will be very complicated, but should be able to be an effective approximator to any realistic dependence.

The fitting of the potentials can be done in a variety of ways which are generally applicable across many types of materials. One popular method in amorphous materials is the Gaussian approximation potential (GAP) method, the first applications of which were to crystalline carbon (as diamond), silicon and iron [38].

**Figure 1.**
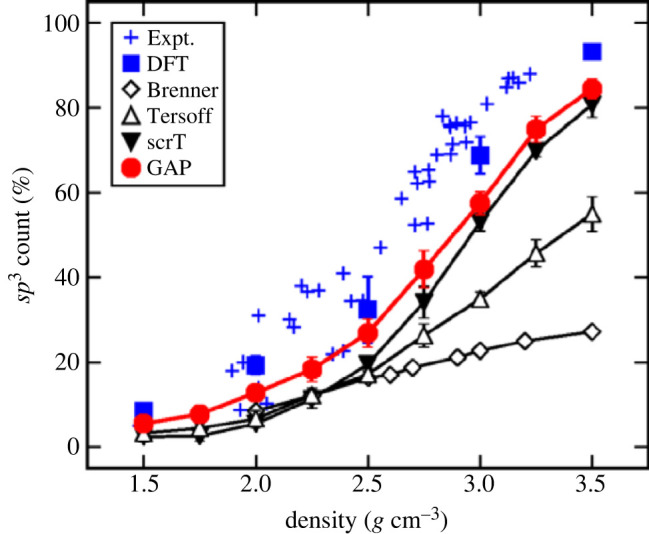
Count of $sp^{3}$ (fourfold coordinated) carbon atoms in quenched a-C structures as a function of density. Ten independent melt-quench cycles were performed at each density for the empirical and GAP models; three independent ones were done for DFT. Error bars represent standard deviations. Lines between data points are only guides to the eye. Reprinted figure from [39]. Copyright (2017) by the American Physical Society. (Online version in colour.)

The first application of these potentials to amorphous materials was to amorphous (and liquid) carbon [39]. The ML potential was seen to outperform other classical non-ML potentials, particularly at reproducing the proportions of $sp^{2}$ and $sp^{3}$ carbon atoms as a function of density, where the results were comparable to DFT and certainly superior to less advanced potentials (see figure 1). The same potential was subsequently applied to studying the deposition of tetrahedral (i.e. $sp^{3}$) amorphous carbon [40], at a range of different deposition energies [41]. An improved potential was subsequently developed by the same investigators [42,43].

The MRO in a-Si is shown experimentally by the presence of a first sharp diffraction peak (FSDP) in the diffraction pattern, a characteristic sign of MRO and one which is often difficult to reproduce in computer modelling. An ML potential was able to generate a-Si structures from a melt-quench trajectory at slower cooling rates than DFT simulations, with structures and properties in good agreement with experiment, including the height of the FSDP [44], allowing for thorough analysis of individual atomic environments and energies [45] and elucidating the structural and electronic transitions that occur as amorphous silicon is put under pressure [46]. The FSDP present in amorphous phosphorus (a-P) is also reproduced by an ML potential fitted to that material [47,48].

Other than the amorphous phases of single elements, ML potentials have also been applied to phase-change materials (PCM), which are a sub-group of chalcogenide glasses, which crystallise very quickly, typically on the scale accessible even to DFT MD simulations, and reversibly, with large property differences between the amorphous and crystalline states [49]. An ML potential has been applied to the prototypic PCM, ${\mathrm{Ge}}_{2}{\mathrm{Sb}}_{2}{\mathrm{Te}}_{5}$ [50], allowing simulations of many thousands of atoms, much bigger than those performed by previous DFT work [49], and revealing more details about the MRO on the length scales at which the crystallization takes place, and the precursors to this crystallization.

Although ML potentials have had some notable successes, enthusiasm for them must be tempered, at least for the moment. It is important that the training set contains the appropriate inputs, as ML potentials, as with all fitting methods, are more likely to fail when extrapolated to structural configurations which do not appear in the trainingset [51].

The materials studied above are either single elements, or comprise elements of similar electronegativities. This means that the bonding is essentially completely covalent, and the contribution of long-range electrostatic forces is negligible. The fitting of these potentials is usually done to data taken at the short range (e.g. neighbours and nearest neighbours in the case of the smooth overlap of atomic positions kernel, as used in the GAP method [45]) and long-range forces are neglected. As these forces are not in the training set to fit the potential, the final potential is not necessarily able to represent them well. This presents a problem for a great many amorphous materials, e.g. most oxides, nitrides and sulfides, as ionic forces play a large part in their structure. These forces are given by Coulomb’s law, in which the force due to two charges drops off as 1/r, where r is the interatomic distance, which can lead to extended chemical order over many tens of Angstroms [24], and which is neglected by the current methods of fitting ML potentials. This can be most clearly seen in a recent work [52] on fitting an ML potential to the prototypic glass-former ${\mathrm{SiO}}_{2}$, in which the properties of the various crystalline phases are well reproduced, but the FSDP in amorphous ${\mathrm{SiO}}_{2}$ is not. This problem does not arise for all amorphous oxides, however, as the structures of amorphous ${\mathrm{HfO}}_{2}$ [53] and TiO${\;}_{2}$ [54], including the FSDP, are well represented by ML potentials.

### (c) Radiation damage and very large simulations

One well-known application of glass is as a long-term storage material for nuclear waste. Nuclear waste remains radioactive over very long timescales (greater than $10^{5}\;\mathrm{years}$) and anything in which the waste is stored will be continuously bombarded by radiation for this length of time and must remain stable under this bombardment to be a viable storage material. Amorphous materials are good candidates for long-term storage because their disordered structure is thought to be less affected by high-energy radiation than crystals; indeed, the effect of radioactive bombardment on crystals is often to create structural defects and ‘amorphize’ the material.

Computer simulation can be used to study the effect of radioactive bombardment on glasses, although there are two methodological challenges. Firstly, due to the very high energies of the recoil atom following decay, a very large simulation cell is needed to capture its trajectory fully, typically $10^{8}$ atoms or larger. This is computationally very expensive, far beyond the reach of DFT, and so classical empirical potentials are needed. Secondly, the energy of the recoil atom is so high that, in its vicinity, the interatomic distances can become very small, far smaller than the distances at which the interatomic potential is fitted, or works well. So, the conventional potential usually has to be coupled to a potential accurate at very short range, such as the ZBL potential [55].

**Figure 2.**
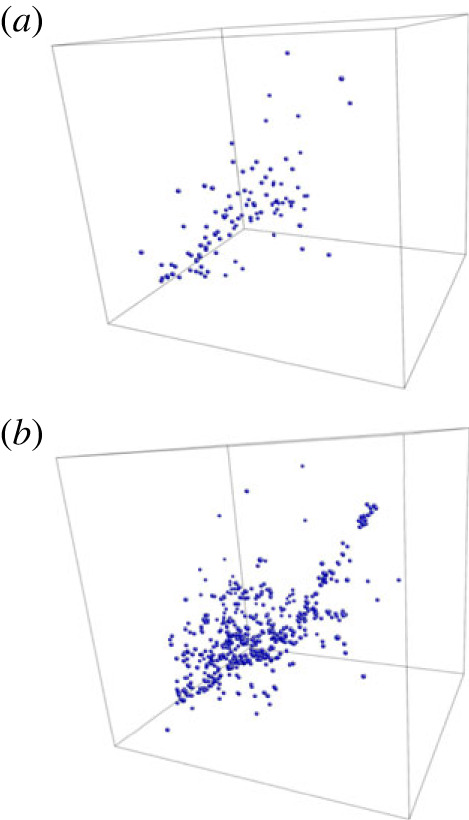
Positions of all newly created three coordinated Si–O atoms inside the simulation cell for (*a*) crystalline and(*b*) amorphous zircon after six overlapping collision cascades. Reprinted from [56] under the terms of the Creative Commons Attribution 4.0 licence. (Online version in colour.)

These techniques have recently been applied to understanding the structure of amorphous zircon (ZrSiO${\;}_{4}$) under radioactive bombardment, with simulation cells containing more than ten million atoms [56,57]. It is clear from this work that the amorphous state spreads the damage due to the bombardment over a larger number of atoms than the crystalline state (figure 2), and that this is not visible from experimental quantities like the pair-distribution function g(r) and more detailed analysis of coordination statistics are needed to characterize the damage fully. The structure continued to evolve during the trajectory of the simulation, and the final structure—and ultimately the question of whether or not these materials are useful to encapsulate nuclear waste—is still open.

### (d) Clustering in phosphate glass: a cautionary tale

It is also instructive to consider where DFT simulations can lead us astray, and here I highlight an example from my own work.

Certain compositions of glass are bioactive, that is, they undergo chemical reactions when implanted into the body. The most well-studied of these glasses is the silicate-based 45S5 Bioglass [58] which undergoes a series of reactions after implantation and subsequently bonds chemically to hard and soft tissues, including human bone [59,60]. Phosphate glasses are also bioactive, and degrade quickly, making them potentially useful as drug delivery materials and in other tissue engineering applications [61,62]. Both silicate- and phosphate-based bioactive glasses can be synthesized containing ions which are useful therapeutically [62,63]. The inclusion of these ions changes the structure and properties of the glass, including the reactions it undergoes after implantation, and there has been considerable effort in attempting to uncover how the inclusion of these ions changes the bioactivity; much of this effort has been computational [5,64–66], and has included the use of HPC computing through DFT-based MD simulations [17,67–73].

When therapeutic ions are included in the glass, there is no requirement that they are distributed uniformly throughout the material, and atomic clustering is thought to have deleterious effects on the bioactivity [64]. Fluorine has applications in dentistry, and when included in bioactive silicate-based glasses, its preference to bond to network-modifying ions (e.g. sodium and calcium) leads it to cluster in regions which subsequently become rich in fluoride ions, and also leave regions which are rich in network-forming ions (silicate in this case), which may affect the bioactivity [74,75]. Although the models produced by DFT simulations are too small to see clustering directly, the local bonding which leads to clustering was clear from DFT simulations of these glasses [76,77].

**Figure 3.**
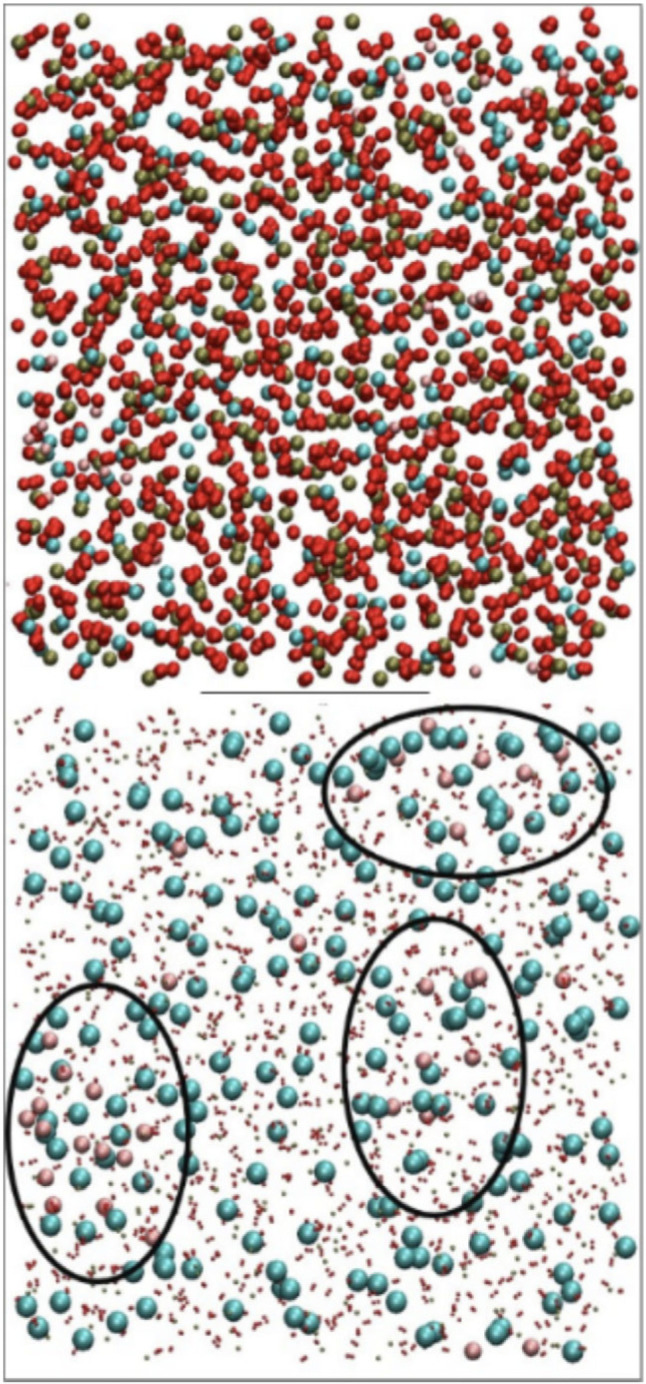
View of a representative fluoridated phosphate glass composition (top) with shrunk oxygen and phosphorus atoms and clusters highlighted (bottom) at 300 K. The colours are: phosphorus (green), oxygen (red), calcium (blue) and fluorine (pink). Reprinted from [79] under the terms of the Creative Commons Attribution 4.0 licence. (Online version in colour.)

It was natural to extend this work to phosphate glasses, and DFT simulations of small (approx. 200–300 atoms) models were performed [78]. By contrast to silicate glasses, it was impossible, when looking at the local environments of the phosphorus atoms, to discern any preferential bonding to network modifiers or otherwise, and the conclusion was drawn that fluoridated phosphate glasses would not suffer any deleterious changes to their bioactivity due to clustering. Later work used empirical potentials which allowed for larger models and showed the opposite to be true: just as for silicate glasses, fluoridated phosphate glasses show clear clustering of fluoride ions present in the glass [79] (figure 3). It seems likely that a combination of the fast cooling rate and the small size of the models in the DFT simulations led us initially to the wrong conclusions.

### (e) Combined simulation and NMR

Solid-state NMR experiments are a powerful technique toward understanding the structure of glass, and can be particularly useful when combined with complementary computer simulations. It is of course possible to conduct separate NMR experiments and computer simulations, and analyse the results jointly, and this has been effective at unravelling even complicated glass structure (as an example, see [80–82] investigating different connectivities and clustering in a multicomponent glass), but this can usually be done with classical potentials and so does not typically require HPC resources, thus falling outside the scope of this review.

**Figure 4.**
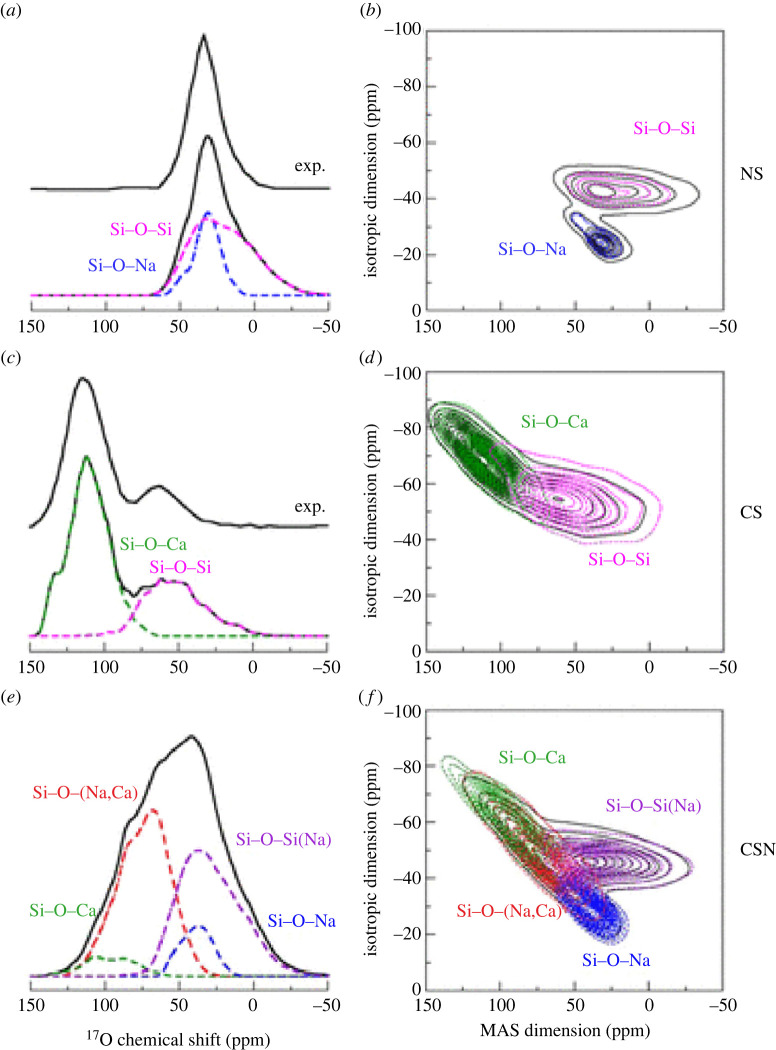
Computed ${\;}^{17}O$ MAS and 3QMAS spectra of sodium silicate (NS), calcium silicate (CS) and sodium calcium silicate (CSN) glasses. The spectra of the different O sites are highlighted in colour. The experimental ${\;}^{17}O$ MAS spectra taken from the literature are also reported for specific compositions. Modifier cations surrounding BOs are reported in square brackets, while those surrounding NBOs are in parentheses. Reprinted with permission from [87]. Copyright (2012) American Chemical Society. (Online version in colour.)

Direct computation of the NMR spectrum can be done using the GIPAW method [83], which rapidly became an important tool for the interpretation of NMR spectra [84], including that of oxide glasses of several types: silicates, borates, germanates, phosphates and aluminates [85–87]. Different values of n in the $Q^{n}$ distribution can be resolved which provides vital information about the connectivity of the glass, and the use of two-dimensional spectra can provide even more precise detail about the interconnectivity of specific different pairs of ions, e.g. the differences between Si–O–Si and Si–O–Na [86] (figure 4).

As above, a note of caution must be sounded: some models investigated in this way are constructed with classical potentials during the melt-quench, and then relaxed with DFT afterwards. This leads to models which have medium-range structural properties, for example, NC, which are given by the classical potentials. In other words, while the DFT relaxation should lead to accurate structures at the short range, it does not correct any inaccuracies due to the classical potential at medium-range length scales. The overall electronic structures, and hence NMR spectra, might not, therefore, be correct.

## 5. Conclusion

The computational study of amorphous materials is extremely important in helping to understand their structure and properties. These models are typically prepared by mimicking the experimental melt-quench procedure in the computer through a long-time MD simulation. There are a variety of choices about how the interatomic forces can be represented, and there is a trade-off between the accuracy of this representation, and computational time and expense.

The simulations which require HPC resources are typically those using either very accurate simulations, usually DFT, or those looking at very large models. One example of a very large model is the use of MD to investigate radiation damage in zircon glass, a possible material for nuclear waste encapsulation. This glass seems to be more able to absorb radiation than crystalline materials, but the simulations are still uncertain as to its ultimate fate.

The extent to which amorphous materials can trap holes and electrons has been extensively investigated, as many devices contain a thin layer of amorphous oxide, and careful simulation reveals that the traps are often deeper in amorphous materials than in crystalline ones. Another application of glass is in drug delivery, and careful re-evaluation of existing DFT simulations of fluoridated phosphate glass with larger models corrected previous errors and showed that these materials might not be as suitable for implantation as first thought.

Methodological improvements in the simulation of amorphous materials is a very active field of research. The development of interatomic potentials fitted by machine learning could offer a very promising route to cheaper but accurate simulations; but these are not yet completely applicable to materials with long-range ionic bonding, which includes almost all oxides. Extensive links between simulation and NMR spectra are an excellent example of using complementary experimental and simulation techniques to understand the behaviour of specific materials.

## Data Availability

This article has no additional data.

## References

[1] Elliott SR. 1990 Physics of amorphous materials, 2nd edn. London, UK: Longman.

[2] Richet P (ed.). 2021 Encyclopedia of glass science, technology, history, and culture. New York, NY: Wiley.

[3] Zachariasen WH. 1932 The atomic arrangement in glass. J. Am. Chem. Soc. **54**, 3841-3851. (10.1021/ja01349a006)

[4] Christie JK, Brauer DS. 2017 The role of fluoride in the nanoheterogeneity of bioactive glasses. Phys. Chem. Glasses **58**, 180-186.

[5] Christie JK, Cormack AN, Hanna JV, Martin RA, Newport RJ, Pickup DM, Smith ME. 2016 Bioactive sol–gel glasses at the atomic scale: the complementary use of advanced probe and computer modeling methods. Int. J. Appl. Glass Sci. **7**, 147-153. (10.1111/ijag.12196)

[6] Machon D, Meersman F, Wilding MC, Wilson M, McMillan PF. 2014 Pressure-induced amorphization and polyamorphism: inorganic and biochemical systems. Prog. Mater. Sci. **61**, 216-282. (10.1016/j.pmatsci.2013.12.002)

[7] Frenkel D, Smit B. 2002 Understanding molecular simulation: from algorithms to applications, 2nd edn. New York, NY: Academic Press.

[8] Martin RM. 2008 Electronic structure: basic theory and practical methods. Cambridge, UK: Cambridge University Press.

[9] Deringer VL, Caro MA, Csànyi G. 2019 Machine learning interatomic potentials as emerging tools for materials science. Adv. Mater. **31**, 1902765. (10.1002/adma.201902765) 31486179

[10] Daw MS, Baskes MI. 1983 Semiempirical, quantum mechanical calculation of hydrogen embrittlement in metals. Phys. Rev. Lett. **50**, 1285-1288. (10.1103/PhysRevLett.50.1285)

[11] Daw MS, Baskes MI. 1984 Embedded-atom method: derivation and application to impurities, surfaces, and other defects in metals. Phys. Rev. B **29**, 6443-6453. (10.1103/PhysRevB.29.6443)

[12] Tilocca A, de Leeuw NH, Cormack AN. 2006 Shell-model molecular dynamics calculations of modified silicate glasses. Phys. Rev. B **73**, 104209. (10.1103/PhysRevB.73.104209)

[13] Ainsworth RI, Di Tommaso D, Christie JK, de Leeuw NH. 2012 Polarizable force field development and molecular dynamics study of phosphate-based glasses. J. Chem. Phys. **137**, 234502. (10.1063/1.4770295) 23267491

[14] Tilocca A. 2008 Short- and medium-range structure of multicomponent bioactive glasses and melts: an assessment of the performances of shell-model and rigid-ion potentials. J. Chem. Phys. **129**, 084504. (10.1063/1.2972146) 19044832

[15] Christie JK, Tilocca A. 2010 Aluminosilicate glasses as yttrium vectors for in situ radiotherapy: understanding composition-durability effects through molecular dynamics simulations. Chem. Mater. **22**, 3725-3734. (10.1021/cm100847p)

[16] Lane JMD. 2015 Cooling rate and stress relaxation in silica melts and glasses via microsecond molecular dynamics. Phys. Rev. E **92**, 012320. (10.1103/PhysRevE.92.012320) 26274174

[17] Christie JK, Tilocca A. 2010 Short-range structure of yttrium alumino-silicate glass for cancer radiotherapy: Car–Parrinello molecular dynamics simulations. Adv. Eng. Mater. **12**, B326-B330. (10.1002/adem.200980081)

[18] Vollmayr K, Kob W, Binder K. 1995 Cooling rate dependence of the internal structure of a Lennard-Jones glass. Europhys. Lett. **32**, 715. (10.1209/0295-5075/32/9/003)

[19] Vollmayr K, Kob W, Binder K. 1996 Cooling-rate effects in amorphous silica: a computer-simulation study. Phys. Rev. B **54**, 15808. (10.1103/PhysRevB.54.15808) 9985651

[20] Tilocca A. 2013 Cooling rate and size effects on the medium-range structure of multicomponent oxide glasses simulated by molecular dynamics. J. Chem. Phys. **139**, 114501. (10.1063/1.4821150) 24070291

[21] Thapa R, Drabold DA. 2022 Atomistic simulations of glasses, ch. 2. New York, NY: Wiley.

[22] Wright AC. 1993 The comparison of molecular dynamics simulations with diffraction experiments. J. Non-Cryst. Solids **159**, 264-268. (10.1016/0022-3093(93)90232-M)

[23] Wright AC, Hannon AC, Sinclair RN, M. Atzmon JWL. 1984 The neutron diffraction double-null isotopic substitution technique. J. Phys. F: Met. Phys. **14**, L201-L205. (10.1088/0305-4608/14/10/002)

[24] Salmon PS, Barnes AC, Martin RA, Cuello GJ. 2007 Structure of glassy ${\mathrm{GeO}}_{2}$. J. Phys.: Condens. Matter **19**, 415110. 28192322 10.1088/0953-8984/19/41/415110

[25] Christie JK, de Leeuw NH. 2017 Effect of strontium inclusion on the bioactivity of phosphate-based glasses. J. Mater. Sci. **52**, 9014-9022. (10.1007/s10853-017-1155-x) 32055076 PMC6991965

[26] Konstantinou K, Mocanu FC, Lee T-H, Elliott SR. 2019 Revealing the intrinsic nature of the mid-gap defects in amorphous ${\mathrm{Ge}}_{2}{\mathrm{Sb}}_{2}{\mathrm{Te}}_{5}$. Nat. Commun. **10**, 3065. (10.1038/s41467-019-10980-w) 31296874 PMC6624207

[27] Rimola A, Costa D, Sodupe M, Lambert JF, Ugliengo P. 2013 Silica surface features and their role in the adsorption of biomolecules: computational modeling and experiments. Chem. Rev. **113**, 4216-4313. (10.1021/cr3003054) 23289428

[28] Tielens F, Gervais C, Lambert JF, Mauri F, Costa D. 2008 Ab initio study of the hydroxylated surface of amorphous silica: a representative model. Chem. Mater. **10**, 3336-3344. (10.1021/cm8001173)

[29] McKenna KP, Shluger AL. 2011 Electron and hole trapping in polycrystalline metal oxide materials. Proc. R. Soc. A **467**, 2043-2053. (10.1098/rspa.2010.0518)

[30] Strand J, Kavani M, Gao D, El-Sayed A-M, Afanas’ev VV, Shluger AL. 2018 Intrinsic charge trapping in amorphous oxide films: status and challenges. J. Phys.: Condens. Matter **30**, 233001. 29692368 10.1088/1361-648X/aac005

[31] Mora-Fonz D, Kavani M, Shluger AL. 2020 Disorder-induced electron and hole trapping in amorphous ${\mathrm{TiO}}_{2}$. Phys. Rev. B **102**, 054205. (10.1103/PhysRevB.102.054205)

[32] Mistry MV, Cottom J, Patel K, Shluger AL, Sosso GC, Pobegen G. 2021 Modelling the interactions and diffusion of NO in amorphous ${\mathrm{SiO}}_{2}$. Model. Simul. Mater. Sci. Eng. **29**, 035008. (10.1088/1361-651X/abdc69)

[33] Mora-Fonz D, Shluger AL. 2020 Modeling of intrinsic electron and hole trapping in crystalline and amorphous ZnO. Adv. Electron. Mater. **6**, 1900760.

[34] Olsson E, Cai Q, Cottom J, Jakobsen R, Shluger AL. 2019 Structural, elastic, vibrational and electronic properties of amorphous ${\mathrm{Sm}}_{2}O_{3}$ from Ab initio calculations. Comp. Mater. Sci. **169**, 109119. (10.1016/j.commatsci.2019.109119)

[35] Dicks O, Cottom J, Shluger AL, Afanas’ev VV. 2019 The origin of negative charging in amorphous ${\mathrm{Al}}_{2}O_{3}$ films: the role of native defects. Nanotechnology **30**, 205201. (10.1088/1361-6528/ab0450) 30716723

[36] Strand J, Dicks O, Kaviani M, Shluger AL. 2017 Hole trapping in amorphous ${\mathrm{HfO}}_{2}$ and ${\mathrm{Al}}_{2}O_{3}$ as a source of positive charging. Microelectron. Eng. **178**, 235-239. (10.1016/j.mee.2017.05.012)

[37] Strand J, Kaviani M, Afans’ev VV, Lisoni JG, Shluger AL. 2018 Intrinsic electron trapping in amorphous oxide. Nanotechnology **29**, 125703. (10.1088/1361-6528/aaa77a) 29332843

[38] Bartók A, Payne MC, Kondor R, Csányi G. 2010 Gaussian approximation potentials: the accuracy of quantum mechanics, without the electrons. Phys. Rev. Lett. **104**, 136403. 20481899 10.1103/PhysRevLett.104.136403

[39] Deringer VL, Csányi G. 2017 Machine learning based interatomic potential for amorphous carbon. Phys. Rev. B **95**, 094203. (10.1103/PhysRevB.95.094203)

[40] Caro MA, Deringer VL, Koskinen J, Laurila T, Csányi G. 2018 Growth mechanism and origin of high ${\mathrm{sp}}^{3}$ content in tetrahedral amorphous carbon. Phys. Rev. Lett. **120**, 166101. (10.1103/PhysRevLett.120.166101) 29756912

[41] Caro MA, Csányi G, Laurila T, Deringer VL. 2020 Machine learning driven simulated deposition of carbon films: from low-density to diamondlike amorphous carbon. Phys. Rev. B **102**, 174201. (10.1103/PhysRevB.102.174201)

[42] Rowe P, Deringer VL, Gasparotto P, Csányi G, Michaelides A. 2020 An accurate and transferable machine learning potential for carbon. J. Chem. Phys. **153**, 034702. (10.1063/5.0005084) 32716159

[43] Rowe P, Deringer VL, Gasparotto P, Csányi G, Michaelides A. 2022 Erratum: an accurate and transferable machine learning potential for carbon. J. Chem. Phys. **156**, 159901. (10.1063/5.0091698) 32716159

[44] Deringer VL, Bernstein N, Bartók AP, Cliffe MJ, Kerber RN, Marbella LE, Grey CP, Elliott SR, Csányi G. 2018 Realistic atomistic structure of amorphous silicon from machine learning-driven molecular dynamics. J. Phys. Chem. Lett. **9**, 2879-2885. (10.1021/acs.jpclett.8b00902) 29754489

[45] Bernstein N, Bhattarai B, Csányi G, Drabold DA, Elliott SR, Deringer VL. 2019 Quantifying chemical structure and machine-learned atomic energies in amorphous and liquid silicon. Angew. Chem. Int. Ed. **58**, 7057-7061. (10.1002/anie.201902625) PMC6563111 30835962

[46] Deringer VL, Bernstein N, Csányi G, Mahmoud CB, Ceriotti M, Wilson M, Drabold DA, Elliott SR. 2021 Origins of structural and electronic transitions in disordered silicon. Nature **589**, 59-64. (10.1038/s41586-020-03072-z) 33408379

[47] Deringer VL, Caro MA, Csányi G. 2020 A general-purpose machine-learning force field for bulk and nanostructured phosphorus. Nat. Commun. **11**, 5641. (10.1038/s41467-020-19168-z) 33122630 PMC7596484

[48] Zhou Y, Kirkpatrick W, Deringer VL. 2022 Cluster fragments in amorphous phosphorus and their evolution under pressure. Adv. Mater. **34**, 2107515. (10.1002/adma.202107515) 34734441

[49] Lee TH, Elliott SR. 2011 Ab initio computer simulation of the early stages of crystallization: application to Ge2Sb2Te5 phase-change materials. Phys. Rev. Lett. **107**, 145702. (10.1103/PhysRevLett.107.145702) 22107213

[50] Mocanu FC, Konstantinou K, Lee TH, Bernstein N, Deringer VL, Csányi G, Elliott SR. 2018 Modeling the phase-change memory material, ${\mathrm{Ge}}_{2}{\mathrm{Sb}}_{2}{\mathrm{Te}}_{5}$, with a machine-learned interatomic potential. J. Phys. Chem. B **122**, 8998-9006. (10.1021/acs.jpcb.8b06476) 30173522

[51] Miksch AM, Morawietz T, Kaestner J, Urban A, Artrith N. 2021 Strategies for the construction of machine-learning potentials for accurate and efficient atomic-scale simulations. Mach. Learn. Sci. Technol. **2**, 031001. (10.1088/2632-2153/abfd96)

[52] Erhard LC, Rohrer J, Albe K, Deringer VL. 2022 A machine-learned interatomic potential for silica and its relation to empirical models. npj Comput. Mater. **8**, 90. (10.1038/s41524-022-00768-w)

[53] Sivaraman G, Krishnamoorthy AN, Baur M, Holm C, Stan M, Csányi G, Benmore CJ, Vázquez-Mayagoitia A. 2020 Machine-learned interatomic potentials by active learning: amorphous and liquid hafnium dioxide. npj Comput. Mater. **6**, 104. (10.1038/s41524-020-00367-7)

[54] Andrade MFC, Selloni A. 2020 Structure of disordered ${\mathrm{TiO}}_{2}$ phases from ab initio based deep neural network simulations. Phys. Rev. Mater. **4**, 113803.

[55] Ziegler JF, Biersack JP, Littmarck U. 1985 Treatise on heavy-ion science, pp. 117-141. New York, NY: Pergamon Press.

[56] Diver A, Dicks O, Elena AM, Todorov IT, Trachenko K. 2020 Evolution of amorphous structure under irradiation: zircon case study. J. Phys.: Condens. Mater. **32**, 415703. 10.1088/1361-648X/ab9f51 32579131

[57] Diver A, Dicks O, Todorov IT, Elena AM, Trachenko K. 2021 Radiation damage effects in amorphous zirconolite. J. Nucl. Mater. **544**, 152654. (10.1016/j.jnucmat.2020.152654)

[58] Hench LL, Splinter RJ, Allen WC, Greenlee TK. 1971 Bonding mechanisms at the interface of ceramic prosthetic materials. J. Biomed. Mater. Res. **5**, 117-141. (10.1002/jbm.820050611)

[59] Hench LL. 1991 Bioceramics: from concept to clinic. J. Am. Ceram. Soc. **74**, 1487-1510. (10.1111/j.1151-2916.1991.tb07132.x)

[60] Hench LL. 1998 Bioceramics. J. Am. Ceram. Soc. **81**, 1705-1728. (10.1111/j.1151-2916.1998.tb02540.x)

[61] Knowles JC. 2003 Phosphate-based glasses for biomedical applications. J. Mater. Chem. **13**, 2395-2401. (10.1039/b307119g)

[62] Lakhkar NJ, Lee I-H, Kim H-W, Salih V, Wall IB, Knowles JC. 2013 Bone formation controlled by biologically relevant inorganic ions: role and controlled delivery from phosphate-based glasses. Adv. Drug Deliv. Rev. **65**, 405. (10.1016/j.addr.2012.05.015) 22664230

[63] Hoppe A, Guldal NS, Boccaccini AR. 2011 A review of the biological response to ionic dissolution products from bioactive glasses and glass–ceramics. Biomaterials **32**, 2757-2774. (10.1016/j.biomaterials.2011.01.004) 21292319

[64] Tilocca A. 2009 Review: structural models of bioactive glasses from molecular dynamics simulations. Proc. R. Soc. A **465**, 1003-1027. (10.1098/rspa.2008.0462)

[65] Tilocca A. 2010 Models of structure, dynamics and reactivity of bioglass: a review. J. Mater. Chem. **20**, 6848-6858. (10.1039/c0jm01081b)

[66] Christie JK, Ainsworth RI, Hernandez SER, de Leeuw NH. 2017 Structures and properties of phosphate-based bioactive glasses from computer simulation: a review. J. Mater. Chem. B **5**, 5297-5306. (10.1039/C7TB01236E) 32264067

[67] Tilocca A, de Leeuw NH. 2006 Structural and electronic properties of modified sodium and soda–lime silicate glasses by car–parrinello molecular dynamics. J. Mater. Chem. **16**, 1950-1955. (10.1039/B517362K)

[68] Tilocca A, de Leeuw NH. 2006 Ab initio molecular dynamics study of 45S5 bioactive silicate glass. J. Phys. Chem. B **110**, 25 810-25 816. (10.1021/jp065146k) 17181225

[69] Tilocca A. 2007 Structure and dynamics of bioactive phosphosilicate glasses and melts from ab initio molecular dynamics simulations. Phys. Rev. B **76**, 224202. (10.1103/PhysRevB.76.224202)

[70] Pedone A, Charpentier T, Malavasi G, Menziani MC. 2010 New insights into the atomic structure of 45S5 bioglass by means of NMR spectroscopy and accurate first-principles simulations. Chem. Mater. **22**, 5644-5652. (10.1021/cm102089c)

[71] Tilocca A, Cormack AN. 2009 Modeling the water–bioglass interface by ab initio molecular dynamics simulations. ACS Appl. Mater. Interfaces **1**, 1324-1333. (10.1021/am900198t) 20355929

[72] Tilocca A, Cormack AN. 2011 The initial stages of bioglass dissolution: a Car-Parrinello molecular-dynamics study of the glass-water interface. Proc. R. Soc. A **467**, 2102-2111. (10.1098/rspa.2010.0519)

[73] Bernardo E, Corno M, Cormack AN, Ugliengo P, Tilocca A. 2014 Probing the fate of interstitial water in bulk bioactive glass by ab initio simulations. RSC Adv. **4**, 36 425-36 436.

[74] Lusvardi G, Malavasi G, Cortada M, Menabue L, Menziani MC, Pedone A, Segre U. 2008 Elucidation of the structural role of fluorine in potentially bioactive glasses by experimental and computational investigation. J. Phys. Chem. B **112**, 12 730-12 739. (10.1021/jp803031z) 18783268

[75] Lusvardi G, Malavasi G, Tarsitano F, Menabue L, Menziani MC, Pedone A. 2009 Quantitative structure–property relationships of potentially bioactive fluoro phospho-silicate glasses. J. Phys. Chem. B **113**, 10 331-10 338. (10.1021/jp809805z) 19572677

[76] Christie JK, Pedone A, Menziani MC, Tilocca A. 2011 Fluorine environment in bioactive glasses: ab initio molecular dynamics simulations. J. Phys. Chem. B **115**, 2038-2045. (10.1021/jp110788h) 21322627

[77] Pedone A, Charpentier T, Menziani MC. 2012 The structure of fluoride–containing bioactive glasses: new insights from first–principles calculations and solid state NMR spectroscopy. J. Mater. Chem. **22**, 12 599-12 608. (10.1039/c2jm30890h)

[78] Christie JK, Ainsworth RI, de Leeuw NH. 2014 Ab initio molecular dynamics simulations of structural changes associated with the incorporation of fluorine in bioactive phosphate glasses. Biomaterials **35**, 6164-6171. (10.1016/j.biomaterials.2014.04.032) 24802671

[79] Touré ABR, Mele E, Christie JK. 2019 Atomic-scale clustering inhibits the bioactivity of fluoridated phosphate glasses. Biomed. Glasses **5**, 76-84.

[80] Mathew R, Stevensson B, Tilocca A, Edèn M. 2014 Toward a rational design of bioactive glasses with optimal structural features: composition–structure correlations unveiled by solid-state NMR and md simulations. J. Phys. Chem. B **118**, 833-844. (10.1021/jp409652k) 24364818 PMC3905695

[81] Mathew R, Turdean-Ionescu C, Stevensson B, Izquierdo-Barba I, Garcìa A, Arcos D, Vallet-Regì M, Edèn M. 2013 Direct probing of the phosphate-ion distribution in bioactive silicate glasses by solid-state NMR: evidence for transitions between random/clustered scenarios. Chem. Mater. **25**, 1877-1885. (10.1021/cm400487a)

[82] Stevensson B, Mathew R, Edèn M. 2014 Assessing the phosphate distribution in bioactive phosphosilicate glasses by ${\;}^{31}P$ solid-state NMR and molecular dynamics simulations. J. Phys. Chem. B **118**, 8863-8876. (10.1021/jp504601c) 24967834

[83] Pickard CJ, Mauri F. 2001 All-electron magnetic response with pseudopotentials: NMR chemical shifts. Phys. Rev. B **63**, 245101. (10.1103/PhysRevB.63.245101)

[84] Charpentier T. 2011 The PAW/GIPAW approach for computing NMR parameters: a new dimension added to NMR study of solids. Solid State Nucl. Magn. Reson. **40**, 1-20. (10.1016/j.ssnmr.2011.04.006) 21612895

[85] Charpentier T, Ispas S, Profeta M, Mauri F, Pickard CJ. 2004 First-principles calculation of ${\;}^{17}O$, ${\;}^{29}\mathrm{Si}$, and ${\;}^{23}\mathrm{Na}$ NMR spectra of sodium silicate crystals and glasses. J. Phys. Chem. B **108**, 4147-4161. (10.1021/jp0367225)

[86] Charpentier T, Menziani MC, Pedone A. 2013 Computational simulations of solid state NMR spectra: a new era in structure determination of oxide glasses. RSC Adv. **3**, 10 550-10 578. (10.1039/c3ra40627j)

[87] Pedone A, Gambuzzi E, Menziani MC. 2012 Unambiguous description of the oxygen environment in multicomponent aluminosilicate glasses from ${\;}^{17}O$ solid state NMR computational spectroscopy. J. Phys. Chem. C **116**, 14 599-14 609. (10.1021/jp304802y)

